# Prognosis of Hippocampal Function after Sub-lethal Irradiation Brain Injury in Patients with Nasopharyngeal Carcinoma

**DOI:** 10.1038/s41598-017-13972-2

**Published:** 2017-10-31

**Authors:** Sharon Chia-Ju Chen, Yoshifumi Abe, Pen-Tzu Fang, Ya-Ju Hsieh, Yung-I Yang, Tzu-Ying Lu, Shoji Oda, Hiroshi Mitani, Shi-Long Lian, Yu-Chang Tyan, Chih-Jen Huang, Tatsuhiro Hisatsune

**Affiliations:** 1 Department of Medical Imaging and Radiological Sciences, Kaohsiung Medical University, Kaohsiung, Taiwan; 2 Department of Medical Research, Kaohsiung Medical University Hospital, Kaohsiung, Taiwan; 3 Department of Integrated Biosciences, Graduate School of Frontier Science, The University of Tokyo, Kashiwa, Japan; 4 Department of Radiation Oncology, Kaohsiung Medical University Hospital, Kaohsiung, Taiwan; 5 Center for Infectious Disease and Cancer Research, Kaohsiung Medical University, Kaohsiung, Taiwan; 6 Graduate Institute of Medicine, College of Medicine, Kaohsiung Medical University, Kaohsiung, Taiwan; 7 Institute of Medical Science and Technology, National Sun Yat-sen University, Kaohsiung, Taiwan; 8 CREST, JST, Saitama, Japan

## Abstract

This work emphasizes the value of assessing hippocampal function by making a timely MRI-based prognosis following a minor dose of hippocampal irradiation after nasopharyngeal carcinomas (NPC) radiotherapy. A quasi-experiment with case-control design and functional assessments (e.g., neuroimaging analysis with fMRI) was conducted to assess hippocampal function after radiotherapy. We delivered 70 Gy of irradiation to nasopharyngeal carcinomas by 6MV helical radiotherapy and collected data from twenty NPC patients and 24 healthy age-matched subjects. Inevitably, hippocampi also received an average dose of 6.89 Gy (range, 2.0–14 Gy). Seed-based functional connectivity of the hippocampus was applied to estimate the cognitive alteration by time before, one month, and four months after irradiation. Afterward, longitudinal-and-cross-sessional statistical inference was determined with time-dependent measurement analysis of variance (ANOVA) with controlled covariance. Over time, there were longitudinal changes in the functional connectivity of hippocampal-related cortices, including the right middle frontal lobe, left superior temporal lobe, and left postcentral gyrus. The findings indicate the presence of functional plasticity, demonstrating how minor irradiation affects functional performance during the early delayed phase of irradiation-induced brain injury.

## Introduction

Nasopharyngeal carcinomas (NPCs) are highly prevalent in southern Asia. As surgical resection is difficult due to the proximity of NPCs to the hippocampus, brain stem, and cerebral arteries, treatment typically involves radiotherapy^1,2^. However, irradiation inevitably exposes brain tissue leading to both imminent tissue death^3^ and progressive cognitive dysfunction^4,5^. The causative mechanisms of cognitive impairment following irradiation are unknown, but three factors are thought to interact and contribute to cognitive impairment after radiotherapy: (1) microenvironment alteration, including endothelial cell malfunction, oligodendrocyte loss, demyelination, white matter necrosis, and inflammatory response^6^; (2) synaptic plasticity changes^7,8^; and (3) disruption of neurogenesis^9–11^.

In addition to the potential impairment of cognitive function due to damaged adult neurons, the deficit of neuronal stem cells produced in the dentate gyrus of the hippocampus^12,13^ would affect cognitive function of the brain. In general, differentiated neuronal stem cells are used to replace damaged neuronal cells to repair cognitive function. However, because neuronal stem cells are highly sensitive to radiation, radiation-induced apoptosis of neuronal stem cells reduces hippocampal neurogenesis^14–16^ and worsens cognitive function^9,17^. Given the sensitivity of neural stem cells to radiation, the direction of therapeutic irradiation is subjectively considered in the case of impairment of hippocampal-related cognitive function^18,19^, with progressive necrosis increasing the severity of impairment^6^. According to the guidelines of the Radiation Therapy Oncology Group (RTOG)^20^, the deposit dose at the hippocampus should be as low as possible. Dose constraints to the hippocampus can minimize radiation-induced cognitive impairment^21,22^. Although this excludes the lethal damage to neurons, there remains the consideration of a stochastic effect in the sub-lethal stage, which might cause mild or recoverable cognitive impairment. Under such circumstances, neuronal precursors are able to generate continuously in the hippocampus^12,13^, usually allowing cognitive function to recover^23^. To date, only studies in animals have shown functional recovery over four months after radiation exposure^8^ but it hasn’t been found in human studies.

Neurophysiological examination^6,24^ and functional neuroimaging^25^ are useful for assessing cognitive function. Functional magnetic resonance imaging (fMRI) can not only detect functional changes (the activated intensity in brain regions) over time^26–28^, but also assess functional connectivity between brain regions (the connected intensity between brain regions)^29–31^, providing information about the functional networking, including the strength and direction of the connection, and the population of neural activity^32^. This functional connectivity has emerged as a means to assess brain neuroplasticity function^17,30^. The neuroplasticity is reflected on the combined outcome of the intrinsic neuronal coupling in the absence of experimental disturbance and the task-sensitive coupling due to extrinsic influence by experimental manipulation or intervention^33^.

We aimed to explore the functional alteration of the hippocampus following NPC radiotherapy in three time-dependent sessions, before, and at one, and four months after radiotherapy. By analyzing the alteration of the functional connectivity to the hippocampus with repeated measurement ANOVA analysis and regression analysis, we found that irradiated NPC patients might suffer from impaired hippocampal function with minor dosage to the hippocampus (2.0–14 Gy); however, its function is recoverable within four months after irradiation. This study was a quasi-experiment with case-control design and included conducting functional assessments (e.g., neuroimaging analysis with fMRI). Our work emphasized the value of timely prognosis regarding hippocampal function following low-dose irradiation of the hippocampus in NPC patients.

## Results

### Participants’ demography

**Figure 1:**
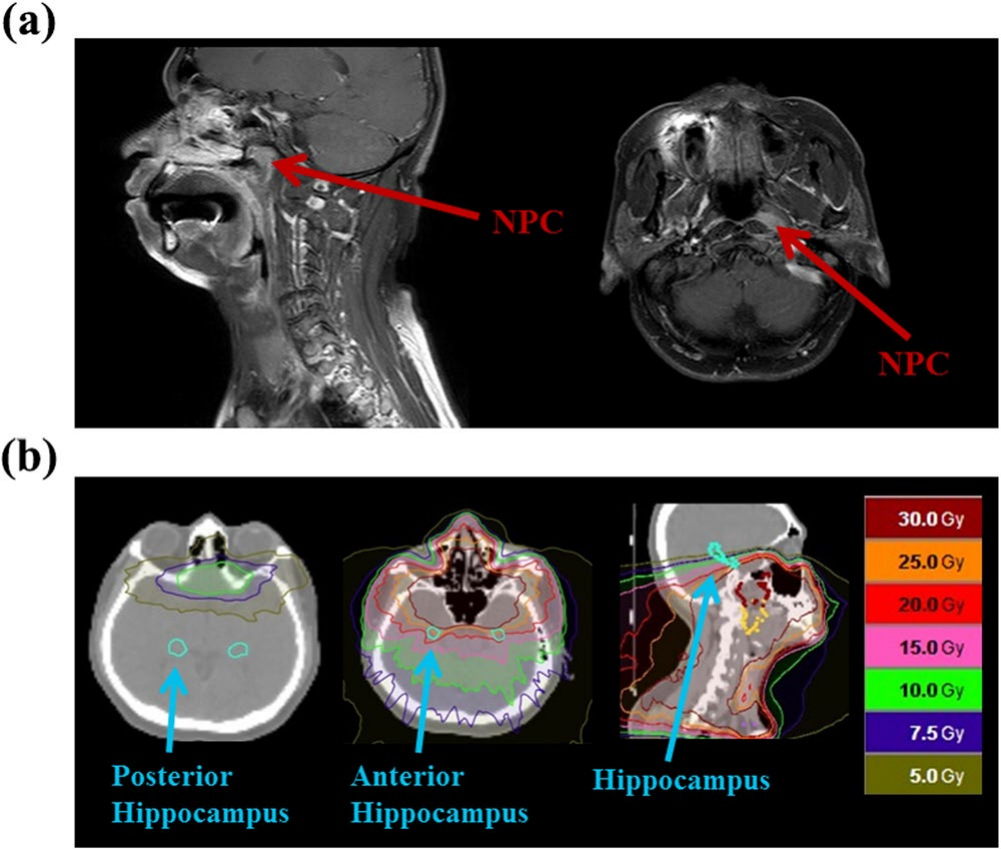
Dosimetric distribution in radiotherapy for nasopharyngeal carcinoma. (**a**) Sagittal and axial magnetic resonance imaging images were used to locate the tumor. (**b**) Overlay of an isodosimetric map on representative axial and sagittal computed tomography images showing the dose-dependent effect of radiation at the near temporal, frontal, and anterior hippocampal areas. The deposit dose at the hippocampus was 2–14 Gy, depending on the distance from the tumor.

In NPC radiotherapy, the hippocampus was dosed mainly at the anterior side due to the input direction of irradiation beam, with doses around 20 Gy close to the hippocampus (within 1 cm) as shown in Figure 1. Table 1 summarizes the demographic information for the 20 patients (15 males; age, 48.32 ± 2.29 years denoting “mean ± standard error of the mean”) and 24 age-matched healthy controls (10 males; age 46.61 ± 3.20 years denoting “mean ± standard error of the mean”). There was no difference in age between two groups (*p*-value* = *0.60). The cancer stages ranged from stage 1 to stage 4 and the average volume at the planning target volume with high risk (PTV-h) was 97.01 ± 11.21 ml denoting “mean ± standard error of the mean”. Because the location of NPC is almost in the middle line of body, the symmetric distribution of the isodose curve was observed in the hippocampi so we adopted the average dose received by the hippocampus as an effective dosimetric parameter (Dose at Hippo.). The mean dose of the hippocampus over patients was 6.89 ± 0.73 Gy (range from 2–14 Gy) denoting “mean ± standard error of the mean”. The influences caused by other clinical confounders such as active smoking, alcohol drinking, diabetes, hypertension and depression were ignorable in our group inference in that we has considered them as controlled variables in the statistical analysis. Besides, the less prevalence of the clinical confounders in our data has limited influence on the reasoning of group inference.

**Table 1:** Demographic and Clinical Information of Patients with Nasopharyngeal Carcinoma.

	Control	NPC	Statistics (*p*-value)^c^
Subjects (n)	24	20	
Sex (male/female)	10/14	15/5	
Age (years)	46.61 ± 3.20	48.32 ± 2.29	0.61
Education (years)	15.50 ± 0.28	14.40 ± 0.56	
Smoke (n)	0	6^d^	
Alcohol (n)	0	3^e^	
Betel nuts (n)	0	1^f^	
Depression (n)	0	0	
Diabetes (n)	0	2	
Hypertension (n)	2	3	
Stage^a^ (stage 1/2/3/4)	—	6/5/6/3	
PTV-h (ml)	—	97.01 ± 11.21	
Dose at Hippo^b^ (Gy)	—	6.89 ± 0.73	

^a^Chemotherapy was applied for advanced disease (stage > 2), at 30 mg/m^2^ per week.

^b^Dosimetry was averaged over both hippocampi.

^c^Two-sample *t*-test with significant value (*p*-value < 0.05) was used to test the difference between groups. “*” denotes *p*-value < 0.05.

^d^About 15 cigarettes a day on average prior to the diagnosis of NPC.

^e^About 300 c.c. of beer per day.

^f^About 20 betel nuts per day prior to the diagnosis of NPC.

The values denoted in count number for item of subjects, sex, education, smoke, alcohol, betel, depression, diabetes, hypertension and cancer stage; the others are presented in mean ± standard error of the mean (SEM).

*Abbreviations:* Hippo, hippocampus.

Routinely, the follow-up check 12 months after the radiotherapy was performed. All patients did not report their cognitive impairment in the daily life and there was no temporal necrosis found on their MRI neuroimaging as well.

### Altered connectivity of the hippocampal network (Hippo) after irradiation

Group inference of functional connectivity for each session was obtained by one-sample *t*-test at the level of statistical significance for functional activation (*p*-value < 0.001) and cluster size constraint (*p*-value < 0.01) using AlphaSim. Overviewing the maps across three sessions, although the location of activated regions did not obviously vary in three sessions, the connected intensity between hippocampus and other brain regions decreased by one month and increased by four months after irradiation. The connection information of the activated areas can be differentiated from the color density in Supplementary Figure S1 or from the statistical measurements (maximum *t*-value in the cluster and the cluster size) in Supplementary Table S1(a) and (b). In contrast, the connectivity to the PCC in the three sessions did not express the affected fluctuations induced by irradiation in the sessions as well as the responses in the normal group. The results of PCC connectivity to the DMN network is shown in Supplementary Figure S2 and Supplementary Table S1(c). Here, PCC network was selected as the reference condition of the brain default mode^31,34^.

Within the group testing, a paired two-sample *t*-test (*p*-value < 0.05), plus AlphaSim cluster constraint (*p*-value < 0.05) was performed to test the differences between each of the two sessions for the NPC group. Supplementary Figure S3 and Supplementary Table S2 show the significant decrease in hippocampal connectivity one month after irradiation in the left inferior frontal cortex and insula, which increased bilaterally four months after radiotherapy in the bilateral inferior frontal cortex, right middle frontal cortex, and left superior temporal lobe. In contrast, PCC connectivity was not altered globally one month after radiotherapy but was prominent enhanced in the superior temporal lobe by four months.

With the results in one condition or the differences in two conditions, we observed that the post-irradiation functional plasticity on connection intensity within hippocampus network was altered along with time but this phenomenon was not observed in the PCC network.

### Longitudinal analysis of altered connectivity of the hippocampal network (Hippo)

**Figure 2:**
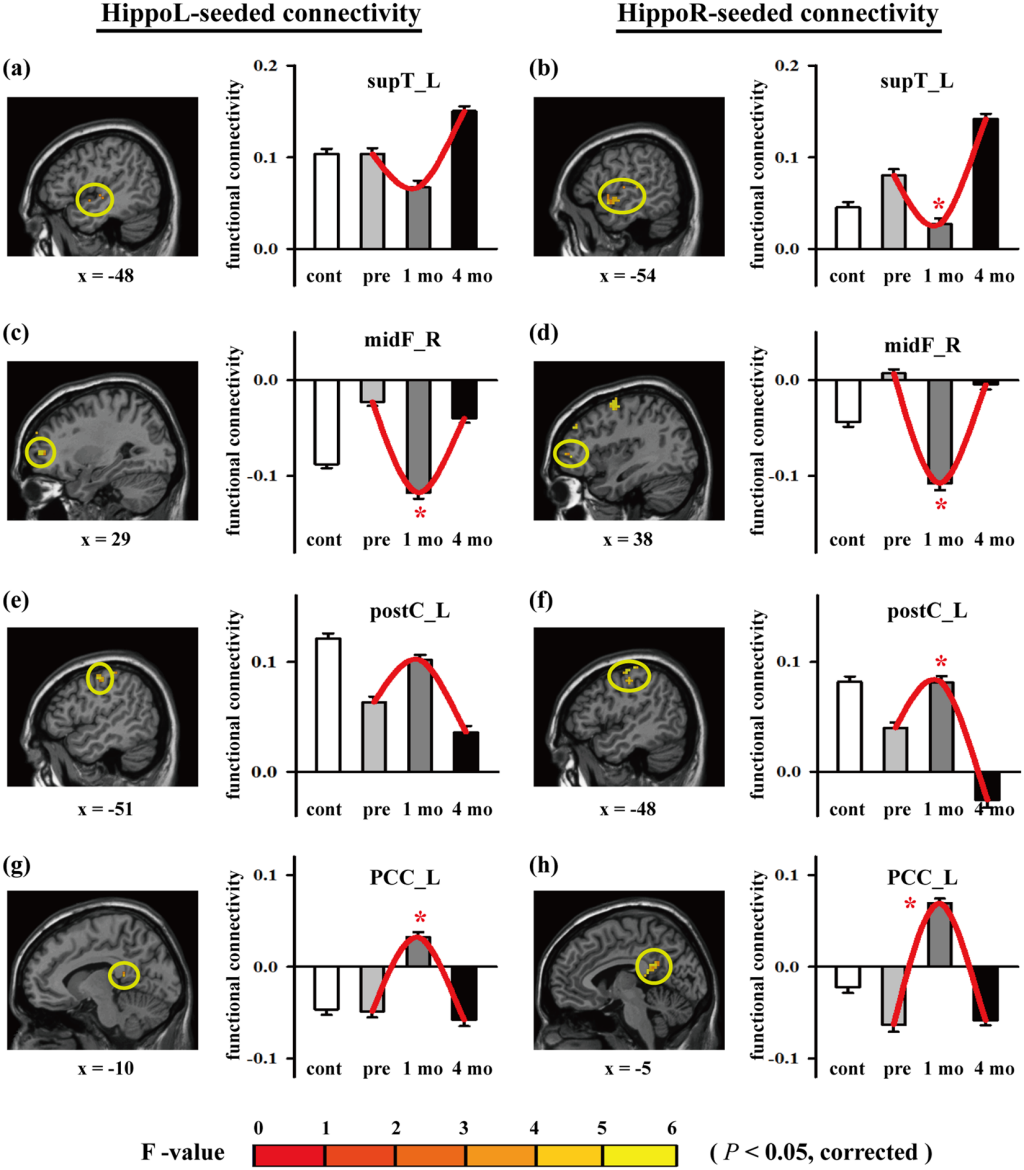
Longitudinal analysis after repeated measurement analysis of variance. Activation maps demonstrating the activated response at interest areas, including the supT_L, midF_R, postC_L, and PCC_L (*p*-value < 0.05). Bar plots presenting functional connectivity across subjects at interest areas for controls and patients with nasopharyngeal carcinoma in three sessions (pretreatment [pre], 1 mo., and 4 mo.). “*” indicates statistical significance. *Abbreviations:* HippoL, left hippocampus; HippoR, right hippocampus; supT_L, left superior temporal lobe; midF_R, right middle frontal lobe; postC_L, left postcentral gyrus; PCC_L, left postcingulate cortex; norm, controls; NPC, nasopharyngeal carcinoma; ROI, region of interest.

To examine the functional deviation caused by irradiation, variance analyses, such as ANOVA, by setting the “pre” condition as the covariate, was performed over three sessions satisfied with statistical constraints for activation and cluster size (both *p*-value < 0.05) (Table 2). For each activated region, we categorized the tendency of connection intensity over three sessions in increasing monotonic, decreasing monotonic, open-up parabola (OU), and open-down parabola (OD). Of the 40 activated regions, four regions were selected for further evaluation: the left superior temporal lobe (supT_L), right middle frontal cortex (midF_R), left postcentral gyrus (postC_L), and left PCC (PCC_L). The first two regions are memory-related areas^17^ and the last two are default-related areas^31^ for the demostration of longitudinal response. The activation maps in Fig. 2 show the activated voxels that passed the statistical constraints. The bar plots illustrate the connection intensity of hippocampal connectivity in the three sessions. The supT_L and midF_R tended to have the OU parabola while the other two regions tended to have the OD parabola when compared with controls. These opposite tendencies between the first two and the last two regions were in alignment with the findings of the previous studies^31,34^. On the other hand, no significant differences (*p*-value > 0.05) were found when the variation of PCC connectivity was tested for the given regions (Supplementary Figure S4), except for the supT_L, indicating that functional connectivity in the PCC network was not significantly altered by radiotherapy under such circumstances.

**Table 2:** Longitudinal analysis with analysis of variance and covariance.

Seed	Region(s)	side	type^a^	vox	F_max_	x	y	z	side	type^a^	vox	F_max_	x	y	z
HippoL-seeded connectivity	Angular	L	OD	56	6.7	47	25	35							
	Cuneus								R	OU	39	7.2	27	14	35
	Fusiform	L	OD	116	18	41	41	13	R	OD	64	6.3	18	35	15
	infO								R	OD	23	5.4	21	12	24
	infT								R	OU	38	7.9	15	44	12
	Insula								R	OU	27	11	18	47	27
	Lingual								R	OU	97	7.8	29	21	25
	MCC	L	OU	74	8.6	34	48	37							
	midF	L	OD	179	13	43	46	45	R	OU	83	7.9	22	60	27
	midO								R	OU	40	9.1	16	19	36
	midT_Pole	L	OD	16	7.5	45	47	15							
	paraHippo	L	OD	15	5.6	40	35	19	R	OD	39	5.1	24	35	19
	PCC	L	OD	28	3.5	34	29	29							
	postC	L	OD	87	8.6	46	35	47							
	Precuneus	L	OD	68	9.2	36	21	35							
	Putamen	L	OD	17	4.3	37	44	28							
	supO								R	OU	86	10	23	16	35
	SupraMarg								R	OU	55	5.3	10	37	34
	supT	L	OU	28	5.4	50	45	24							
	supT_Pole								R	OU	60	9.9	16	50	20
HippoR-seeded connectivity	Angular	L	OD	56	9.3	45	23	36							
	Cuneus								R	OU	39	14	24	17	41
	Fusiform		OD	64	7.9	21	32	20							
	Lingual								R	OU	97	6.4	26	22	24
	MCC	L	OU	74	11	34	30	38							
	midF	L	OD	179	11	43	60	35	R	OU	83	11	19	43	45
	midO	L	OU	88	11	46	17	34							
	midT_Pole	L	OD	16	6.1	47	46	15	R	OU	12	8.1	12	45	20
	midT								R	OU	93	9.3	15	21	29
	paraHippo	L	OD	15	7.6	40	33	19	R	OD	39	6.7	22	37	17
	PCC	L	OD	28	7.8	34	27	33							
	postC	L	OD	87	11	48	38	41	R	OD	57	8	15	36	46
	Precuneus	L	OD	68	8.1	34	24	32							
	Putamen	L	OD	17	5.8	37	44	28							
	Rolandic_Oper	L	OU	35	4.8	50	46	27							
	sup-medF	L	OD	37	7.6	34	60	39	R	OD	25	5.7	28	58	38
	supO								R	OU	86	8	23	16	38
	SupraMarg								R	OU	55	9.8	14	34	34
	supT	L	OU	28	5.3	49	45	21	R	OU	12	5.3	9	36	31
	supT_Pole								R	OU	60	8.1	16	48	20

For each activated region, the tendency of functional connectivity across the three sessions was labeled by parabolas that opened either upward or downward after analyzing repeated measurement ANOVA analyses with the AlphaSim cluster extension. The pretreatment (pre) data was used as the covariate to adjust the 1 mo. and 4 mo. data. Normal subjects were used for comparison. ^a^OU, parabolas open upward; OD, parabolas open downward.

### Validation between hippocampal functional connectivity and dosimetric parameter

**Figure 3:**
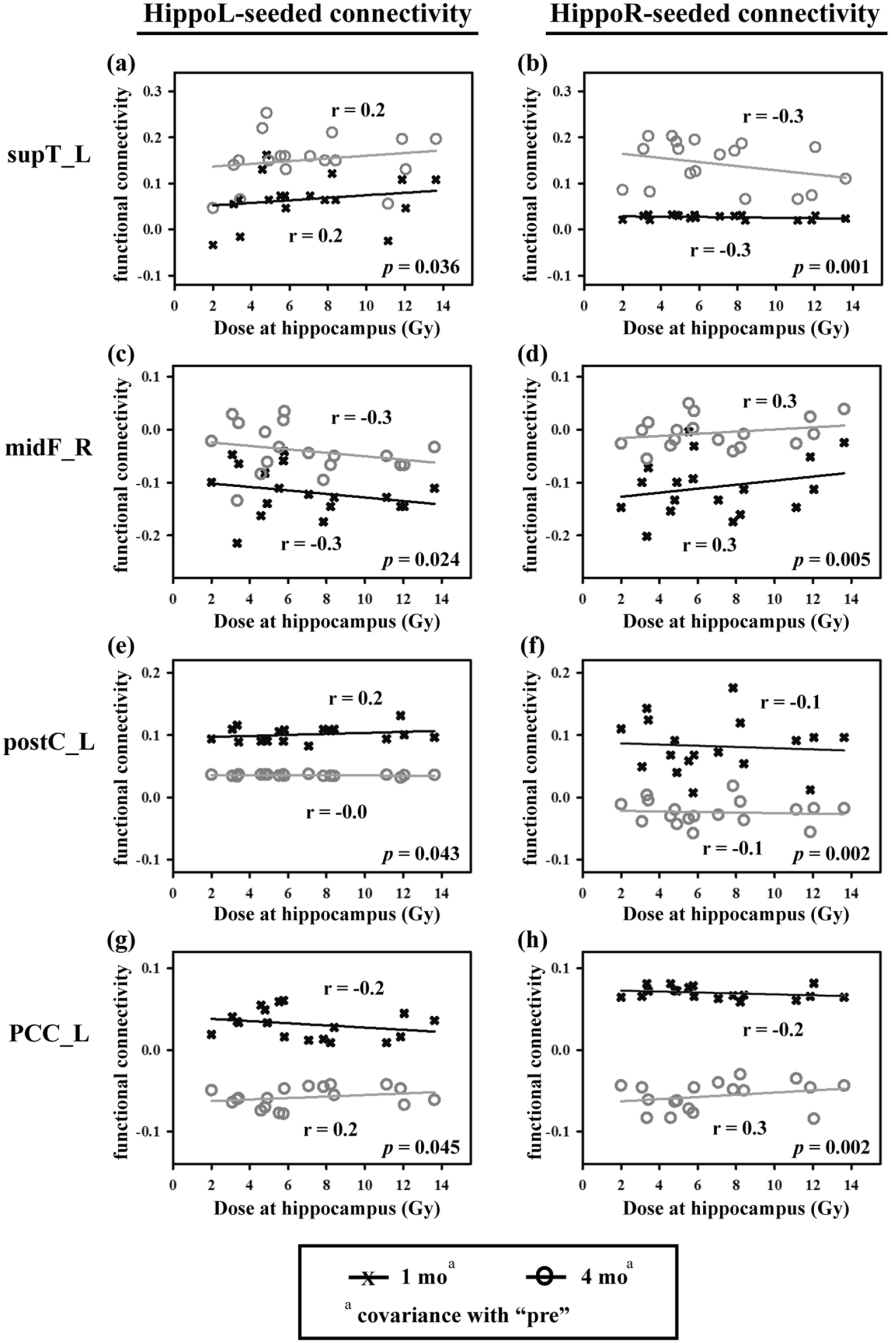
Scatter plots of hippocampal dosimetry and functional connectivity. Connectivity of selected regions (four interest areas) for each seeded region of the left and the right hippocampi (hippoL and hippoR). All data points were corrected at baseline by their “pre” data points. Corrected “1 mo.” and “4 mo.” were tested with paired two-sample *t*-test and *p*-values are labeled on the right side. *Abbreviations:* HippoL, left hippocampus; HippoR, right hippocampus; supT_L, left superior temporal lobe; midF_R, right middle frontal lobe; postC_L, left postcentral gyrus; PCC_L, left postcingulate cortex; ROI, region of interest.

We built up the relationship between deposit dose at hippocampus and hippocampus connectivity with scatter plots for the four given brain regions and examined the relations with regression models for “1 mo” and “4 mo” sessions (after removal of the covariate) (Fig. 3). Two memory-related areas, supT_L and midF_R showed significantly correlation in the hippocampal connectivity, and this relationship was invert dependence with the deposit dose at hippocampus; however, there were only mild correlations in the DMN-related regions, postC_L and PCC_L.

The above resultant observations only demonstrated the intensity of the relationship between two variables; however, their relationship might be strongly interacted with each other. Thereafter, we furthered the regression model test on the dependent variable (functional connectivity) and the two independent variables (deposit dose at hippocampus and follow-up session). Also, we assessed the partial correlation between the dependent variable and each independent variable further by removing the effect of the controlled covariate as shown in Table 3. Four regions all passed the model testing (*p*-value < 0.05) that was mainly attributed to the irradiation application (“follow-up session” variable) because only this variable consistently passed the marginal testing (*p*-value < 0.05). In addition, the partial correlation coefficient is able to interpret the unique contribution to dependent variable form the independent variable, which reflected the degree of association between functional connectivity and each independent variable. For example, the dose at the hippocampus showed a negligible influence in the regions of the supT_L, the postC_L and the PCC_L but exerted a significant effect in the regions of the midF_R, which are highly related to memory function. Under such circumstance, the irradiation application played a very important role in the modulation of brain function.

**Table 3:** Regression model between the dependent variable (functional response) and independent variables (deposit dose at hippocampus and follow-up session).

Y(D/T)^a^	β_std_^b^	*t*-value	Partial C.C.^c^	Model (F-value)
supT_L	0.2/0.7	1.2/5.0^d^	0.2/0.7^d^	13.3^d^
midF_R	−0.2/0.7	−1.6/4.9^d^	−0.3^d^/0.7^d^	15.2^d^
postC_L	<0.1/−1.0	0.8/−19.4^d^	0.1/−1.0^d^	188.1^d^
PCC_L	<0.1/−1.0	−0.2/−17.9^d^	<0.1/−1.0^d^	160.1^d^

^a^Y: the region of interest; D: deposit dose at hippocampus; T: the follow-up session. the statistic model: Y = X(D, T).

^b^All βcoefficients are presented after standardization.

^c^The partial correlation coefficient.

^d^Statistical significance (*p*-value < 0.05) for model test and marginal test.

*Abbreviations:* supT_L, left superior temporal lobe; midF_R, right middle frontal lobe; postC_L: left posterior central gyrus.

**Figure 4:**
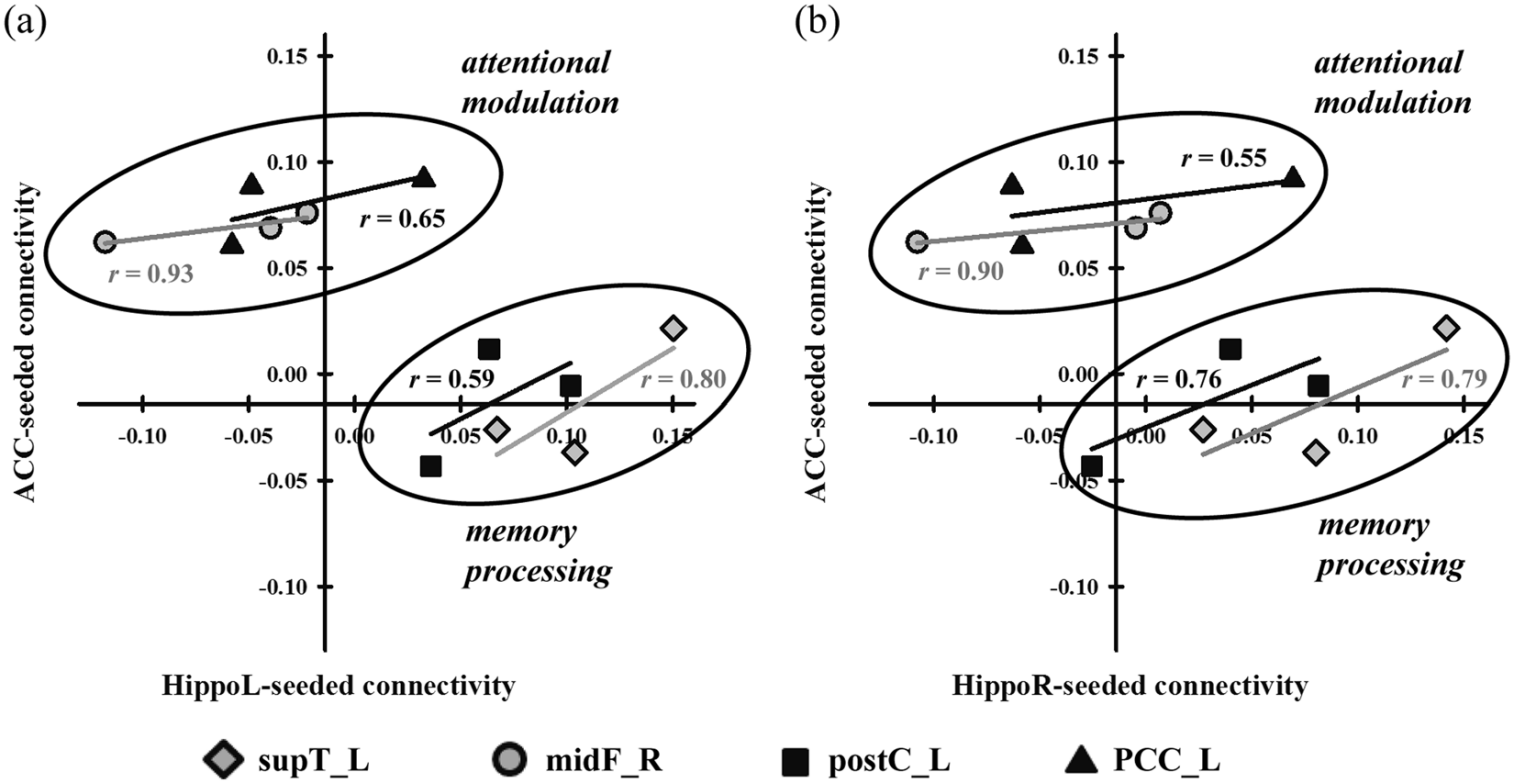
The scatter plot between Hippocampus-seeded connectivity and ACC-seeded connectivity across sessions. The connection intensity of four respective regions in two networks, hippocampal and ACC functional connectivity, was represented. The linear regression lines were drew across three sessions for each region and remarked their correlation coefficient (*r*). These regions were divided into two functional clusters associated with attentional modulation and memory processing. *Abbreviations:* HippoL, left hippocampus; HippoR, right hippocampus; supT_L, left superior temporal lobe; midF_R, right middle frontal lobe; postC_L, left postcentral gyrus; PCC_L, left postcingulate cortex.

Besides, because of the high functional correlation in cognitive performance between memory network and attentional network, we also examined the attentional network by calculating the ACC network and demonstrated the correlation distribution of three sessions with scatter plot between two networks, for assessing the contribution between the hippocampus and the ACC for neurocognitive functions, such as attention and memory processing. In Fig. 4, the four regions, which we depicted in the previous analysis, were grouped into two functional clusters, attentional modulation and memory processing based on which cognitive function was enhanced at the specific region. The supT_L and postC_L were grouped in the memory-related storage and sensory integration^35^. The midF_R and PCC_L were assigned to the attentional-related planning and consciousness^36^.

## Discussion

We investigated whether irradiation affected functional connectivity, especially for the multi-tasking hippocampus, with minor dosimetry for the NPC^24^. To clarify the relationship between irradiation dose and hippocampal-related networking over time, we used fMRI neuroimaging assessment (functional connectivity to the hippocampus) to compare controls with patients receiving different deposit dose at hippocampus. Even minor hippocampal irradiation (2–14 Gy) downgraded its functional connectivity with other regions of the brain, but this recovered by four months after radiotherapy.

In radiation-induced brain injury, early delayed responses are less well understood than late-delayed injury^6,37^. Early delayed injury has been reported to resolve by 1–6 months after irradiation^38^, implying repair of anatomic and functional deficits, including demyelination, somnolence, attention deficit, or short-term memory loss. Through neuroimaging using an fMRI, we observed functional fluctuations to the middle frontal lobe, superior-middle temporal lobe, postcentral gyrus, superior-middle occipital lobe, and lingual gyrus, reflecting plasticity of functional networking within a few months after irradiation. The middle frontal lobe, part of the dorsolateral prefrontal cortex, is involved in attention and working memory^36^, while the superior temporal lobe and postcentral gyrus comprise a mirror neuron system constructed by the accumulation of life experience^35^. Our results revealed an effect of the minor dose during the early delayed injury induced by irradiation. Additionally, consistent with previous studies, the functional expression of the PCC was opposite to that of the tasked functional response^39^. On the other hand, the PCC-seeded connectivity, as shown in Supplementary Figure S3(c), its connectivity of the PCC-related DMN was not significantly changed, indicating that hippocampal-seeded connectivity was more severely affected by radiotherapy than that of the PCC. This relationship between the hippocampus and the PCC may represent an imaging biomarker of functional recovery after cranial irradiation.

Although the mechanism of functional recovery following early delayed phase irradiation-induced injury is poorly understood, some cell and animal studies have shown increased neurogenesis in the dentate gyrus would repair the low-dose effect of irradiation (around 5 Gy) by 3 months. It is possible that neuronal precursors differentiate to replace injured brain tissue and promote functional recovery^15,40^. In Tan’s study^15,40^, they conducted a dose-response quantitative experiment with total single-fraction dose to study the effect of radiation by longitudinally observing Ki67 fluctuation, a marker for proliferating cells, and found the reversible effects on neurogenesis used the doses below 5 Gy but the irreversible effects used the higher doses above 5 Gy. Overall, the low-dose injury of irradiation causes the transient depletion of neurogenesis within 1–3 months but the enhanced compensatory proliferation of precursors happens later. However, to our knowledge, there is no human study or any associated studies about low-dose injury of radiation. The results of our study show improved cognitive function of the hippocampus after radiotherapy in NPC patients. We hypothesize that changes in the hippocampal network are coupled with hippocampal neurogenesis: the sublethal dose to healthy neurons may cause cognitive dysfunction in the early phase of irradiation-induced brain injury, but the regenerated neurons will rapidly repair this dysfunction.

Irradiation-induced brain injury is caused by dynamic interactions between multiple cell types^11^, and severe injury might cause permanent late-delayed cognitive impairment by 6 months^1,4^. Current evidence from human studies has confirmed that permanent injury occurs with a high dose (>30 Gy) of whole-brain irradiation. Studies have described this effect following whole-brain irradiation for brain metastasis, glioma, or leukemia^20,22,41^, but required high doses likely to accelerate injury and confound cognitive assessment by introducing dynamic factors that contribute to poor cognitive function (e.g., vascular malformation, oligodendrocyte loss, demyelination, white matter necrosis, and inflammatory response)^7,11^. The assessment of injury induced by high-dose irradiation, therefore, will have been biased and will neglect the mild self-recovery mechanism. This is because lethal damage has effectively removed any possibility of neuronal recovery since it leads to a chain reaction of tissue death including progressive impairment of cognition. Given that NPC results in only partial brain irradiation, this setting provides a simplified environment for assessing cognitive alteration in early delayed injury^21,22^. We were able to detect regional functional changes under such comparatively unmixed circumstances, thereby providing evidence that the functional connectivity of impaired networks recovered by four months. This might have been due to neurogenesis following minor irradiation during the early delayed phase of brain injury. Similar observations have been made in animal studies, with evidence of a temporary reduction in cognitive function after radiation treatment and subsequent recovery within a few months^8^.

### Limitation and future work

Considering the difficulty of follow-up examination in NPC patients, we performed no long-term follow-up beyond six months. Although we observed functional recovery and migration of the hippocampal network, we were not able to confirm whether this recovery was permanent from the present work^6,37^. There is also possible that the impact on the brain tissues outside the hippocampus by the low-dose irradiation delivered using helical tomotherapy could also lead to decline in the cognitive impairment. In addition, due to the specific treatment requirements of NPC patients, we did not vary the treatment methods in the experimental design. However, it would be interesting to see if similar results were obtained following treatments other than helical tomotherapy and for tumors other than NPC. Given that both the intensity of connectivity between regions and the dosimetric distribution will change over time potentially inducing different levels of cognitive decline, further investigation is needed. Finally yet importantly, although the recruitment of NPC patients was difficult to some extent, a larger sample size would definitely improve the statistical power of the data analysis. In this work, our data was conformed to an alternative normal distribution (skewness of population was below twice the standard error) and the type-I error rate was below 5% in the ANOVA analysis, compensated the sample size obstacle.

## Conclusions

Our goal was to examine the cognitive recovery consistently presented as our outcome during our assessment of neuronal functional connectivity. Through our longitudinal and cross-sessional analysis, our work might provide therapeutic criteria for assessing irradiation-induced brain injury by preventing the gradual increase in reactive oxygen species, especially for sub-lethal irradiation damage.

## Materials and Methods

### Study design

This prospective study was conducted between May 2012 and July 2014 after approval of the protocol by the ethics committee of Kaohsiung Medical University Hospital (KMUH-IRB-20120255). All methods were used and experiments performed, in accordance with the Declaration of Helsinki. Participants gave their informed consent prior to participation.

All acquired resting-state functional data was analyzed the functional connectivity of the hippocampus and the posterior cingulate cortex (PCC) across three sessions before and after radiotherapy. Longitudinal comparison across sessions was made by repeated measurement analysis of variance (repeated measurement ANOVA).

### Participant recruitment

We recruited 23 patients with NPC who received fractionated partial brain radiotherapy and 24 age-matched controls to enroll in this study. The common inclusion criteria were: (a) right-handedness and (b) no history of neurological or psychiatric illness. In addition to the common criteria, the patient group necessarily met the following additional criteria: (a) definite biopsy-confirmed NPC; (b) no distant metastasis (M0); and (c) no previous head or neck radiotherapy. All patients have confirmed their MRI neuroimaging prior to radiotherapy and one-year follow-up after irradiation by their radiologist for that they did not have temporal necrosis with which the cognitive function would impaired around hippocampus.

All participants were provided the appropriate information about the nature, purpose, and specific details of this study. Data was excluded for excessive motion during fMRI acquisition or if the participant was considered unable to provide informed consent. Three patients were excluded from data analyses: two because of excessive head motion during fMRI acquisition and one because of the low intelligence score recorded.

### Radiotherapy settings

All patients were treated with definitive intensity-modulated radiotherapy by 6MV Helical Tomotherapy (Accuray Incorporated, Sunnyvale, CA) at a guideline of Department of Radiation Oncology, Kaohsiung Medical University Hospital. The dose distribution of irradiation was estimated and calculated by a Pinnacle platform (version 8.0, ADAC Laboratories, Milpitas, CA) according to the mean attenuation of tissue(s) in Hounsfield Units after the computed tomography simulation with a guide of the vendor (Philips, Brilliance 16 CT scanner). The target tumor received the clinically prescribed dose, 70 Gy for NPC cancer. Doses to organs at risk (OAR), such as optic nerve, brain stem, spinal cord, optic chiasm, mandible, parotid glands, eyes, oral cavity, and larynx were limited to below the tolerant dose constraints according to the recommendations of The National Comprehensive Cancer Network (NCCN) and the Radiation Therapy Oncology Group (RTOG-0225).

Tumor boundaries and critical organs were contoured manually. The full dose of radiation exposure was decided according to the risk assessment of the biopsy proven cancer stage from the near to the distant tumor margin and the critical value of the organ. Radiation was delivered with 70, 63, and 56 Gy to the planning target volume with high risk (PTV-h), PTV with middle risk (PTV-m), and PTV with low risk (PTV-l) zones, respectively, at a fractional dose of 2 Gy per weekday for up to 35 days. Given the consideration of conservative treatment, chemotherapy was routinely administered to patients at cancer stage 2 or above (2–3 cycles of 70 mg/m^2^ cisplatin on day 1 and 500–1000 mg/m^2^ 5-fluorouracil on days 2–5, every three weeks) recommended by RTOG-0225 report. Cancer stage is defined by a gross consideration of tumor size, lymph nodes affected and metastases based on the result of pathological biopsy, classified cancer progression from stage 0 to stage 4 and determined by the American Joint Committee on Cancer (AJCC).

### Acquisition of fMRI data

All patients were scanned in a 3.0-Tesla MRI scanner (Signa HDx, GE, Milwaukee, WI, USA) before, and at one and four months after radiotherapy. Controls were scanned once in the same scanner. The fMRI data were obtained using echo planar imaging with the following parameters: repetition time, 2000 ms; echo time, 35 ms; flip angle, 80°; field of view, 220 × 220 mm; acquisition matrix, 64 × 64; slice thickness, 3.4 mm; slice number, 28; and volume number, 180. Because of scanning during the resting state, participants were asked to lie quietly, close their eyes, and think of nothing during data acquisition for 6 min. After fMRI scanning, none of the subjects reported that he/she had fallen asleep during scanning. Structural data was acquired using a T1 image, 3D-anatomical image dataset (3D-SPGR sequence), set as follows: repetition time, 2.5 ms; echo time, 4.38 ms; flip angle, 8°; field of view, 240 × 240 mm; acquisition matrix, 256 × 256; slice thickness, 1 mm; and slice number, 124.

### Resting-state data analysis

All fMRI data were analyzed with the toolkit of Data Processing & Analysis of Brain Imaging (DPABI, http://rfmri.org/DPABI)^37^, an extension of Statistical Parametric Mapping (SPM8) (http://www.fil.ion.ucl.ac.uk/spm). The first 10 volumes of each subject’s dataset were discarded for signal stability, leaving 170 volumes. The data were first shifted into time synchronization within a volume, and then the volumes were realigned against head movement during scanning. Next, the structural image and realigned functional images were normalized onto the Montreal Neurological Institute (MNI) standard template^42^ in 3 × 3 × 3 mm^3^ resolution according to diffeomorphic anatomical registration, using exponentiated Lie algebra. Finally, normalized functional images were spatially smoothed with a Gaussian kernel function of 4-mm full-width at half-maximum (FWHM). After preprocessing, systematic drift was removed from the functional images by the linear detrend method and later the spontaneous resting frequency reserved within 0.01–0.1 Hz with a band-pass filter.

To calculate the functional connectivity of the hippocampal network, a seeded spherical 5-mm region of interest (ROI) was placed at the right and left anterior hippocampus for the dentate gyrus^12,23,43^. The centers of the seeded ROIs in MNI coordinates were *x* =  ±24 mm, *y* = −13 mm, and *z* = −20 mm which was identified by a virtual navigation task^44^ and this area was regarded as the location of neurogenesis^12,23,43^. Functional connectivity maps were generated by calculation of correlated intensities between the average time course of the seeded ROIs and other brain areas voxel by voxel. Comparatively, the functional connectivity of the attentional network and the default mode network (DMN) was enclosed by setting two 5-mm radius ROIs at the ACC and the PCC as the reference seeds, centering at (*x*, *y*, *z*) = (−1, 39, 20) and (−1, −53, 26) respectively in MNI coordinates. PCC possess a stable expression of activation while individuals stay at a resting state and thus the response at this region was commonly used as a reference compared to other task-related functional regions^31,34^.

### Statistical inference

Statistical inference was determined through paired two-sample *t*-tests, repeated measurement ANOVA, and linear regression analysis in a three-level hierarchical sense of individual, group, and longitudinal follow-up. First, the correlation intensity of the reference seed was transformed into a z-score by Fisher transformation, and the z-map was used to decide individual statistical inferences and to make further group inferences. At the group level, one-sample *t*-tests were applied to assess the single-session inference over subjects; and, paired two-sample *t*-tests were used to compare the difference between each of two temporal sessions. Accordingly, repeated measurement ANOVA analysis involving a covariate control, the data prior to irradiation, was used to analyze changes along with three sessions. During the statistical comparison, alpha probability simulation (AlphaSim) was used as an additional tool to assess the spatial extent of activation to determine whether activated voxels within a cluster above a given *t*-value were statistically reliable by computing the probability of random noise. The resultant *t*-contrast map was superimposed on the co-registered structural MRI image. As for the application of validation, multiple indicators were compared, including deposit dose at hippocampus, and the alteration of functional connectivity to the hippocampus, using SPSS (IBM SPSS 19.0, Illinois, USA).

## Electronic supplementary material

Supplementary Information
