# Supplementary material for: Prognosis of Hippocampal Function after Sub-lethal Irradiation Brain Injury in Patients with Nasopharyngeal Carcinoma
Source: Sci Rep. 2017 Oct 31;7:14697. doi: 10.1038/s41598-017-13972-2 (PMC5665929; doi:10.1038/s41598-017-13972-2)
Supplement: Supplementary file 1 — Supplementary Information [file 41598_2017_13972_MOESM1_ESM.pdf]

Prognosis of Hippocampal Function after Sub-lethal Irradiation Brain Injury in Patients with Nasopharyngeal Carcinoma

Sharon Chia-Ju Chen, PhD[1,2], Yoshifumi Abe, PhD[3], Pen-Tzu Fang, MD[4], Ya-Ju Hsieh, PhD[1], Yung-I Yang, BS[1], Tzu-Ying Lu, MS[4], Shoji Oda, PhD[3], Hiroshi Mitani, PhD[3], Shi-Long Lian, MD[4], Yu-Chang Tyan, PhD[1,2,5,6,7], Chih-Jen Huang, MD, PhD[4,*], Tatsuhiro Hisatsune, PhD[3, 8,*]

Supplementary Material

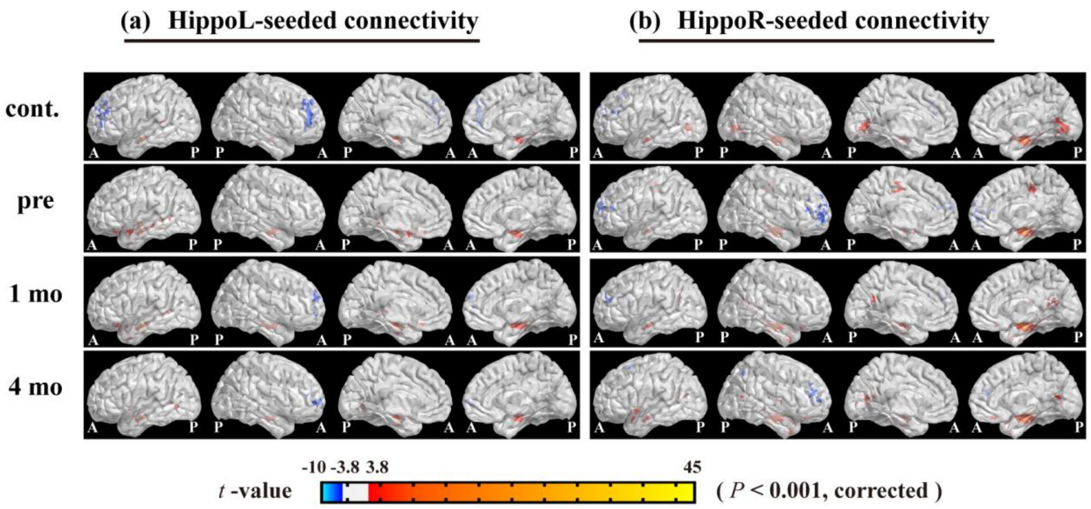

Figure S1. Activation maps of the brain of patients with nasopharyngeal carcinoma compared between normal participants and the right and left hippocampi.

The left column demonstrates the outcome calculated by the seed region of the left hippocampus. The right column demonstrates the outcome calculated by the seed region of the right hippocampus. Each row denotes the patients' results from pretreatment (pre), 1 month and 4 months after treatment (1 mo. and 4 mo.), and normal subjects' results. The statistical threshold was designated p-value < 0.001 after AlphaSim cluster extension criteria under p-value < 0.01. Analysis by one-sample t-tests.

Abbreviations: HippoL, left hippocampus; HippoR, right hippocampus; cont, controls; ROI, region of interest.

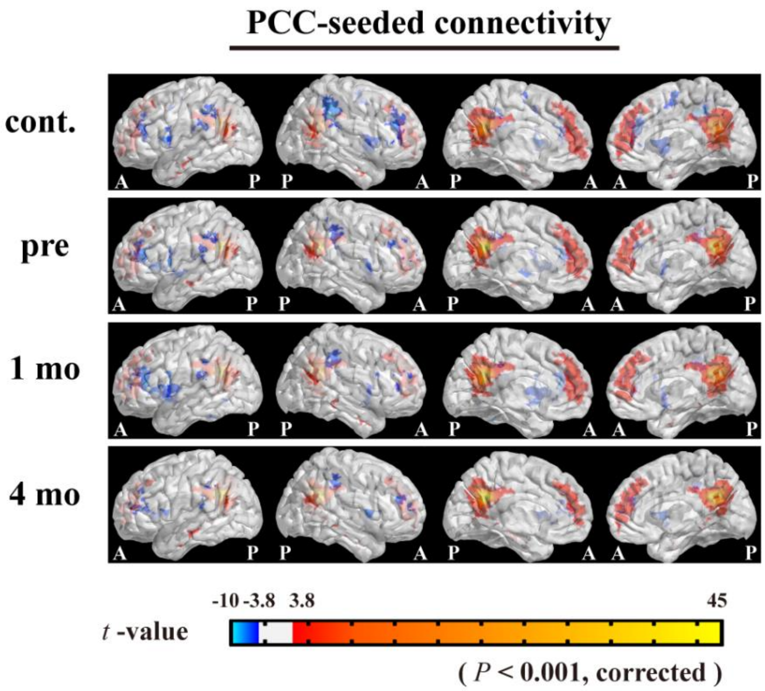

Figure S2. Activation brain maps of NPC patients and normal participants (cont) after one-sample *t*-test analysis. The seed region was set at PCC. Each row denotes pre-treatment (pre), 1 month and 4 months after treatment (1 mo. and 4 mo.), and normal subjects. The statistical threshold was designated under *p*-value < 0.001 for activation and under *p*-value < 0.01 the AlphaSim cluster extension criteria. Data was analyzed by a one-sample *t*-test by setting the seed region at the postcentral cortex (PCC: posterior cingulate cortex).

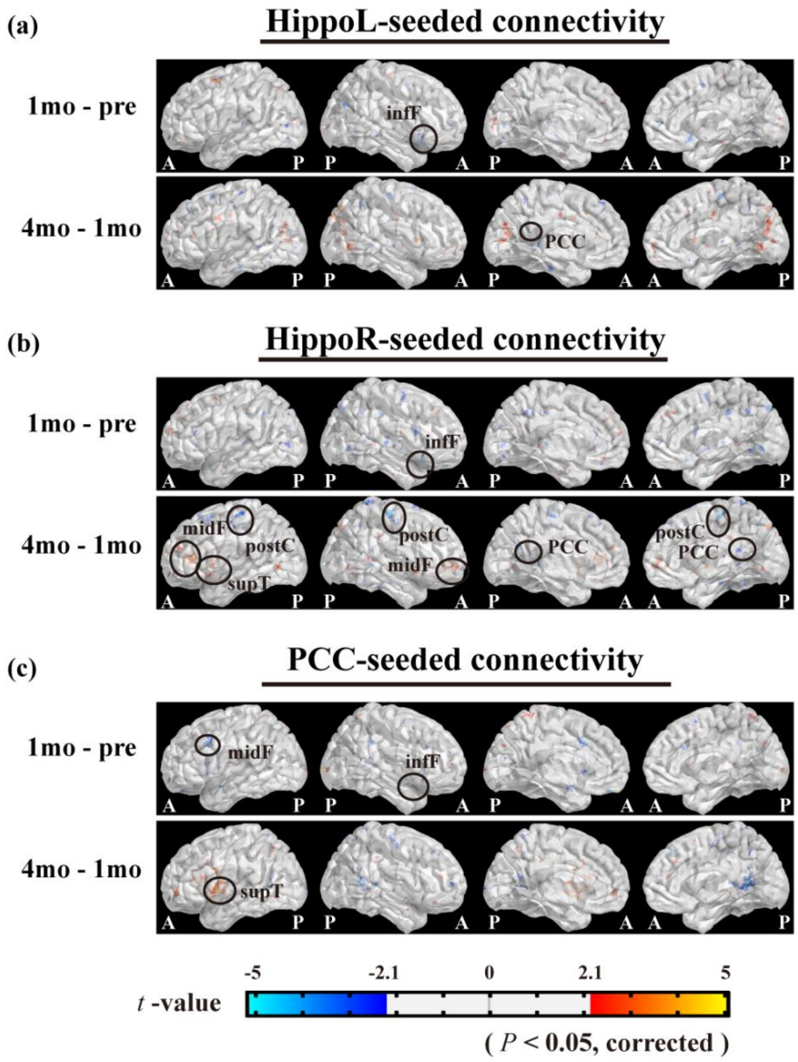

Figure S3. Statistical maps of paired two-sample *t*-tests between the two sessions for each three seeded region of interest (ROI: region of interest) area. The statistical criterion for activation and AlphaSim criterion for cluster size were set at p-value < 0.05.

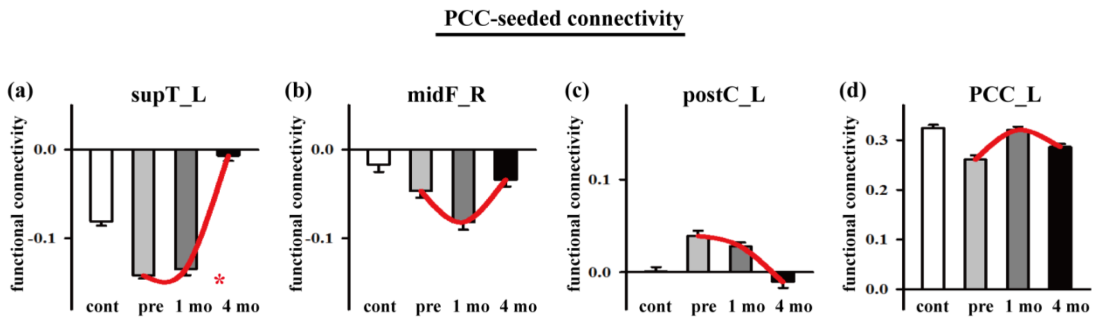

Figure S4. Longitudinal analysis after repeated measurement ANOVA for PCC connectivity. Functional connectivity was analyzed in the same four areas across three sessions. Only the supT_L (left superior temporal lobe) was significantly affected. The statistical criterion was set at *p*-value < 0.05 and cluster size constraint was set at *p*-value < 0.05 after AlphaSim assessment.

Table S1. Within-session seed-connective maps for each session. Three seed ROI areas: (a) HippoL area; (b) HippoR area; and (c) PCC area were used to trace cognitive transition after radiotherapy (one-sample *t*-test; *p*-value < 0.001 error probability for activation; *p*-value < 0.01 for cluster size constraint).

(a)

session	area	side	vox	t_{max}	x	y	z	side	vox	t_{max}	x	y	z
pre	Amygdala	L	20	13.72	-27	-6	-18	R	14	11.73	24	-3	-18
	Fusiform	L	16	16.2	-30	-15	-24						
	Hippo	L	92	48.15	-24	-12	-21	R	37	15.79	24	-6	-18
	infF	L	34	9.31	-24	27	-24						
	midT	L	161	9.03	-63	-15	-9						
	Olfactory	L	11	10.46	-21	6	-18						
	paraHippo	L	51	24.32	-24	-15	-24	R	43	14.87	21	-9	-21
1mo	Amygdala	L	28	11.64	-24	-3	-21						
	Fusiform	L	65	20.79	-30	-15	-24						
	Hippo	L	121	48.63	-27	-12	-21						
	Insula	L	13	6.51	-36	-6	-9						
	Pallidum	L	16	7.95	-18	-3	6						
	paraHippo	L	74	27.47	-27	-15	-24						
	Putamen	L	53	7.41	-21	0	6						
4mo	Amygdala	L	18	11.24	-24	-6	-18						
	Fusiform	L	28	18.91	-30	-15	-24						
	Hippo	L	75	39.15	-24	-9	-21						
	paraHippo	L	48	19.88	-18	-12	-24						
norm	Amygdala	L	25	16.15	-24	-6	-18						
	Fusiform	L	44	22.49	-30	-15	-24						
	Hippo	L	107	55.4	-27	-12	-21						
	infP	L	31	8.79	-48	-24	39						
	midT	L	35	8.84	-60	-42	9						
	paraHippo	L	65	30.42	-24	-15	-24						
	postC	L	99	8.25	-60	-18	36						
	preC	L	25	7.93	-42	-15	57						
	SupraMarg	L	16	8.24	-54	-24	42						
	supT	L	88	8.21	-57	-30	9						

(b)

session	area	side	vox	t_{max}	x	y	z	side	vox	t_{max}	x	y	z
pre	Amygdala							R	19	11.07	21	-3	-18
	Fusiform	L	23	12.49	-30	-21	-27	R	40	11.26	33	-24	-24
	Hippo	L	47	13.72	-24	-9	-18	R	86	38.46	24	-12	-21
	paraHippo	L	42	10.26	-18	-12	-24	R	120	51.02	24	-12	-24
1mo	Amygdala							R	28	10.27	21	-3	-18
	Fusiform	L	49	9.19	-27	-21	-27	R	51	9.35	33	-36	-18
	Hippo	L	56	11.74	-18	-12	-21	R	68	43.57	24	-12	-21
	paraHippo	L	80	12.17	-24	-21	-27	R	138	51.77	21	-12	-21
4mo	Amygdala							R	23	13.53	27	-3	-18
	Fusiform	L	37	8.65	-30	-12	-33	R	10	9.61	27	-27	-21
	Hippo	L	50	12.2	-24	-9	-21	R	78	31.44	27	-9	-21
	paraHippo	L	38	8.82	-18	-12	-24	R	92	29.63	21	-12	-21
norm	Amygdala	L	15	10.72	-24	-6	-18	R	14	11.5	27	-3	-18
	Fusiform	L	25	8.33	-30	-15	-24	R	40	7.06	27	-39	-12
	Hippo	L	66	13.01	-24	-9	-21	R	82	39.59	24	-12	-21

	Lingual	L	82	7.09	0	-75	0	R	75	10.72	9	-78	-12
	orbF							R	30	6.87	24	24	-24
	paraHippo	L	47	8.45	-21	-18	-24	R	106	34.34	24	-12	-24

(c)

session	area	side	vox	t_{max}	x	y	z	side	vox	t_{max}	x	y	z
	ACC	L	103	9.28	0	48	15	R	83	9.85	3	48	24
	Angular	L	122	11.98	-42	-63	24	R	150	13.52	48	-63	24
	Calcarine	L	127	22.7	-3	-63	15	R	110	22.14	3	-60	18
	Cuneus	L	93	26.92	-6	-63	24	R	22	10.29	3	-72	30
	infF	L	297	-3.97	-39	45	6	R	116	5.31	39	18	33
	infP	L	74	-3.97	-57	-33	42	R	56	-3.97	51	-45	51
	infT							R	14	5.86	54	-18	-18
	Insula	L	133	-3.98	-45	-6	0	R	113	-3.98	33	15	12
	Lingual	L	25	9.68	-6	-63	6	R	23	8.08	6	-57	6
	MCC	L	79	23.72	-3	-48	33	R	51	21.23	3	-51	33
	medF							R	34	7.66	3	48	-3
	midF	L	245	8.05	-21	33	42	R	331	8.4	21	30	39
pre	midO	L	50	10.91	-42	-66	24	R	22	11.01	48	-66	24
	midT	L	209	10.02	-42	-63	21	R	264	14.64	51	-60	21
	PCC	L	87	55.54	0	-54	27	R	54	39.6	3	-54	30
	postC	L	10	-3.98	-48	-6	36						
	preC	L	93	-3.98	-42	3	18	R	10	-4.03	57	3	18
	Precuneus	L	304	45.83	0	-57	24	R	274	50.12	3	-54	24
	Putamen							R	26	-4.02	27	12	6
	Rectus	L	14	5.74	-6	60	-15						
	RolOper	L	44	-3.97	-48	6	3	R	68	-3.99	39	-3	15
	supF	L	199	9.77	-15	48	36	R	146	9.63	18	36	39
	supP							R	11	-4.08	45	-45	57
	SupraMarg	L	160	-3.98	-54	-45	30	R	216	-3.99	63	-27	27
	supT	L	137	-3.98	-48	6	-6	R	60	12	51	-57	21
	ACC	L	100	10.65	0	45	15	R	63	10.1	3	45	15
	Angular	L	128	9.85	-45	-72	30	R	149	11.19	51	-60	30
	Calcarine	L	148	23.25	-3	-63	18	R	81	36.96	3	-60	15
	Cuneus	L	110	24.57	0	-66	21	R	51	10.83	3	-72	30
	infF	L	565	-3.99	-45	9	27	R	185	-4.13	51	42	-3
	infP	L	81	-3.98	-39	-51	45	R	12	-3.97	57	-42	48
	infT	L	113	-3.97	-42	-30	-18	R	67	9.53	57	-6	-27
	Insula	L	243	-3.97	-39	12	6	R	92	-3.98	48	3	0
	Lingual	L	36	10.61	0	-63	6	R	47	14.74	9	-51	6
	MCC	L	90	14.37	0	-48	33	R	63	24.55	6	-51	33
1mo	medF	L	26	9.43	0	57	-3	R	25	11.83	3	54	-3
	midF	L	219	-3.97	-48	39	30	R	212	-3.97	33	39	15
	midO	L	47	10.01	-39	-63	24	R	22	7.64	51	-66	24
	midT	L	95	7.85	-48	-63	21	R	271	11.06	54	-3	-27
	PCC	L	91	42.88	-3	-54	27	R	69	37.82	3	-48	27
	Precuneus	L	405	39.74	0	-57	27	R	312	41.08	3	-57	15
	Putamen	L	21	-3.99	-27	6	3						
	RolOper	L	93	-4.08	-60	9	3	R	43	-3.97	57	9	0
	supF	L	137	8.63	-21	39	36	R	120	8.92	21	27	45
	SupraMarg	L	82	-3.99	-60	-27	21	R	115	-3.99	66	-30	27
	supT	L	96	-3.97	-51	-6	3	R	53	-3.97	54	0	-3
	ACC	L	66	10.32	0	48	15	R	73	10.9	3	48	18
4mo	Angular	L	123	16.29	-42	-63	24	R	85	12.89	51	-60	24
	Calcarine	L	107	16.83	-3	-63	18	R	62	18.95	3	-60	18
	Caudate	L	24	-4.11	-15	18	9						

	Cuneus	L	78	22.4	-3	-63	24	R	35	8.23	3	-72	30
	infF	L	142	-3.99	-48	33	15	R	84	-3.97	39	33	15
	infP	L	24	-3.98	-54	-45	39	R	17	-4.07	57	-39	51
	infT	L	54	8.38	-57	-6	-30	R	35	7.38	54	-9	-27
	Insula	L	127	-3.98	-30	15	-6	R	97	-3.98	39	6	-3
	Lingual	L	23	7.27	-3	-63	6	R	25	6.73	9	-45	0
	MCC	L	80	24.04	0	-48	33	R	58	19.25	3	-51	33
	medF	L	18	7.47	0	57	-3	R	23	7.53	3	57	-3
	midF	L	212	-3.97	-42	48	18	R	269	-3.98	36	54	24
	midO	L	24	13.18	-39	-63	24	R	16	7.38	57	-63	24
	midT	L	248	12.33	-42	-63	21	R	196	11.14	54	-60	21
	PCC	L	91	62.5	0	-54	27	R	64	44.43	3	-51	30
	Precuneus	L	303	50.84	0	-57	27	R	251	61.21	3	-54	24
	Putamen							R	39	-4.09	30	6	9
	RolOper							R	46	-4.03	60	9	0
	SMA							R	10	6.35	12	24	51
	supF	L	164	8.02	-18	30	45	R	124	7.18	21	30	45
	supP							R	12	-3.97	54	-33	57
	SupraMarg	L	50	-3.97	-57	-36	36	R	68	-3.97	63	-36	36
	supT	L	15	-4	-66	-36	12	R	16	10.49	57	-60	21
	ACC	L	112	9.15	0	48	15	R	115	9.3	3	45	27
	Angular	L	125	11.51	-48	-63	24	R	72	11.12	48	-60	24
	Calcarine	L	178	20.15	0	-63	15	R	139	26.99	3	-60	18
	Cuneus	L	83	24.99	-3	-63	24	R	27	9.78	3	-72	30
	infF_Oper	L	83	-3.97	-42	6	21	R	52	-3.97	48	12	0
	infF_Orb							R	23	-4.04	57	33	-3
	infF_Tri	L	233	-3.98	-36	33	6	R	180	-3.98	48	36	9
	infP	L	64	-3.98	-57	-51	36	R	116	-3.99	42	-51	48
	infT	L	52	7.41	-54	-9	-27	R	37	6.68	54	0	-33
	Insula	L	112	-3.97	-33	0	15	R	191	-4	39	-3	-9
	Lingual	L	42	11.45	0	-63	6	R	79	21.47	3	-60	6
	MCC	L	120	20.48	0	-48	33	R	76	17.6	3	-48	33
norm	medF_Orb	L	96	7.36	-6	60	-6	R	49	7.05	6	60	-6
	midF	L	121	6.46	-24	18	42	R	425	-3.97	45	48	24
	midO	L	23	9.77	-39	-63	24	R	16	7.67	45	-63	27
	midT	L	234	10.56	-51	-60	18	R	184	14.32	51	-60	21
	PCC	L	93	33.38	0	-54	27	R	67	27.18	3	-57	30
	Precuneus	L	352	33.78	0	-57	24	R	280	34.73	3	-57	21
	Putamen							R	43	-4.02	33	0	6
	RolOper	L	57	-4.09	-42	0	12	R	63	-4	57	3	6
	SMA	L	59	-3.97	0	-12	63	R	138	-3.97	3	-9	51
	supF	L	162	8.17	-12	42	42	R	200	7.48	24	27	48
	supP							R	18	-4.06	42	-51	60
	SupraMarg	L	126	-3.97	-66	-24	30	R	306	-3.98	51	-45	33
	supT	L	29	-3.99	-63	-36	18	R	68	-3.98	54	-36	21

Table S2. Between-session seed-connective maps between two sessions. Three seed ROI areas: (a) HippoL area; (b) HippoR area; and (c) PCC area were used to trace cognitive transition after radiotherapy (two-sample *t*-test; *p*-value < 0.05 error probability for activation; *p*-value < 0.05 cluster size constraint).

(a)

$H_0 < 0$	area	side	vox	t_{max}	x	y	z	side	vox	t_{max}	x	y	z
	Caudate	L	16	3.08	-15	9	9						
	Fusiform	L	26	5.08	-30	-6	-36						
	infF							R	34	-2.11	42	27	-18
	Insula							R	34	-2.12	36	15	-6
1mo-pre	midF	L	87	4.22	-36	9	57						
	paraHippo	L	23	3.03	-24	-9	-30						
	preC	L	10	3.88	-39	3	57						
	Putamen	L	29	3.56	-18	3	9						
	supF	L	42	3.73	-12	48	42						
	Angular	L	74	-2.12	-45	-72	36	R	12	-2.16	48	-60	51
	Calcarine	L	78	3.52	0	-72	15	R	74	3.13	9	-78	3
	Cuneus	L	42	3.36	-12	-81	27	R	88	3.68	15	-84	30
	Fusiform	L	54	-2.11	-33	-24	-18						
	Hippo	L	16	-2.11	-15	-36	9						
	infF	L	16	3.82	-60	9	21						
	infP	L	15	-2.15	-36	-57	45	R	47	-2.13	51	-48	48
	infT	L	27	-2.17	-42	-33	-27						
	Lingual	L	21	2.73	-6	-66	6	R	64	3.4	9	-66	3
	MCC	L	41	3.87	-6	-3	36	R	27	3.23	6	-12	39
4mo –	midF	L	59	-2.13	-36	3	54	R	16	3.71	39	9	42
1mo	midO	L	25	3.62	-24	-75	27	R	14	3.29	30	-78	30
	paraHippo	L	23	-2.13	-27	-30	-15						
	PCC	L	12	-2.15	-9	-51	27						
	postC							R	33	3.97	54	-12	27
	preC	L	76	4.33	-45	-3	27	R	52	3.49	39	3	42
	Precuneus	L	40	-2.14	-18	-48	6						
	RolOper	L	21	3.6	-45	-6	18						
	supF	L	61	-2.11	-15	45	45						
	supO	L	45	3.94	-21	-75	27	R	96	5.38	24	-81	30
	supP	L						R	11	3.38	24	-81	48
	SupraMarg							R	24	3.36	60	-15	27

(b)

$H_0 < 0$	area	side	vox	t_{max}	x	y	z	side	vox	t_{max}	x	y	z
	infF	L	92	-2.11	-51	27	9	R	73	-2.11	63	12	12
	infP	L	16	3.74	-45	-27	48	R	16	3.74	-45	-27	48
	Insula							R	28	-2.12	42	15	-9
	Lingual							R	81	-2.11	24	-60	0
1mo-pre	MCC							R	19	-2.16	6	-24	39
	midF	L	25	3.32	-21	42	33	R	42	-2.11	33	3	57
	postC	L	45	4.03	-42	-30	48						
	preC							R	61	-2.14	39	-9	45
	Precuneus							R	30	-2.13	6	-51	36
	supF	L	32	3.51	-27	48	39	R	21	-2.14	36	-6	66
	Angular	L	78	-2.12	-48	-54	24	R	38	-2.14	51	-57	42
4mo – 1mo	Cuneus	L	47	-2.2	-12	-69	21	R	24	3.51	18	-78	45
	Fusiform							R	63	-2.13	33	-24	-24

	infF	L	281	4.34	-51	33	9	R	110	4.13	39	45	-6
	infO	L	16	3.24	-54	-72	-3						
	infP							R	23	-2.16	45	-57	45
	infT							R	11	-2.12	45	-57	-21
	Insula	L	22	4.22	-39	9	0						
	midF	L	88	4.66	-33	51	30	R	103	4.45	39	54	0
	midO	L	11	2.55	-48	-69	0						
	midT	L	38	3.8	-51	-66	0	R	60	3.72	57	-45	0
	paraHippo							R	51	-2.12	24	-18	-27
	PCC	L	37	-2.13	-6	-45	27	R					
	postC	L	90	-2.13	-39	-33	51	R	73	-2.14	51	-27	57
	preC	L	28	3.51	-45	0	24	R	60	-2.12	33	-21	72
	Precuneus	L	68	-2.12	-6	-54	24						
	RolOper	L	15	3.39	-42	-3	18						
	SMA	L	31	-2.14	-9	18	69	R	12	-2.17	12	24	63
	supF	L	22	-2.13	-18	24	57	R	21	3.27	30	51	9
	supO							R	70	4.34	24	-81	39
	supP							R	42	-2.13	30	-54	69
	SupraMarg							R	59	4.76	57	-33	39
	supT	L	28	3.32	-54	-6	-9						

(c)

$H_0 < 0$	area	side	vox	t_{max}	x	y	z	side	vox	t_{max}	x	y	z
1mo-pre	Amygdala	L	13	-2.17	-21	-3	-18						
	Calcarine	L	40	4.14	-15	-102	-3						
	Caudate	L	23	-2.22	-9	21	0	R	30	-2.11	12	21	-6
	Cuneus	L	34	5.13	0	-81	33	R	30	3.34	12	-72	33
	Heschl							R	10	3.13	48	-18	6
	infF	L	106	-2.16	-51	21	21	R	27	-2.16	36	21	24
	infT							R	33	3.91	48	-45	-6
	Insula	L	26	-2.13	-36	12	12	R	14	4.61	36	-18	6
	midF	L	86	-2.12	-36	18	39	R	19	-2.11	30	18	39
	midO	L	29	3.24	-9	-96	0	R	28	4.98	27	-96	3
	midT							R	27	4.36	48	-45	-3
	paraHippo	L	10	-2.55	-15	3	-24						
	postC							R	25	3.06	42	-39	63
	preC	L	11	-2.12	-54	12	30	R	20	2.45	36	-27	63
	Precuneus	L	33	3.35	-6	-54	69	R	22	3.74	3	-57	69
	Putamen	L	21	-2.12	-18	15	-9						
	Rectus	L	27	-2.12	-3	54	-21						
	RolOper							R	10	2.67	42	-24	15
	supF	L	18	-2.16	-18	60	-12						
	supO	L	14	3.05	-9	-99	12	R	10	3.89	24	-99	3
4mo – 1mo	Angular							R	52	-2.11	42	-69	33
	Calcarine	L	113	-2.11	-21	-60	9	R	76	-2.12	15	-60	18
	Cuneus	L	16	-2.16	-12	-63	21						
	Fusiform							R	83	2.17	21	-33	-15
	Hippo							R	16	-2.24	24	-39	6
	infF	L	436	5.2	-57	24	18						
	infP	L	75	3.86	-45	-48	45						
	infT	L	30	3.92	-45	-57	-6						
	Insula	L	66	5.02	-36	0	15						
	Lingual	L	16	-2.23	-9	-60	0	R	39	-2.12	18	-45	0
	midF	L	91	3.64	-33	18	33						
	midO	L	21	-2.15	-12	-102	3						
	midT	L	22	3.32	-54	-63	3						

	paraHippo							R	32	-2.11	33	-39	-12
	postC	L	12	3.34	-48	-3	15	R	38	-2.13	51	-15	57
	preC	L	68	4.15	-39	3	18	R	49	-2.12	48	-9	60
	Precuneus	L	18	-2.12	0	-57	18	R	73	-2.12	12	-45	15
	RolOper	L	102	6.12	-57	3	3						
	supF	L	18	2.75	-24	54	3						
	supP							R	66	-2.12	24	-72	60
	supT	L	68	6.39	-54	6	-3						

Abbreviation

ACC	Anterior Cingulate Cortex
Hippo	Hippocampus
infF	Inferior Frontal Cortex
infF_Oper	Inferior opercularis Frontal Cortex
infF_Orb	Inferior orbital Frontal Cortex
infF_Tri	Inferior triangular Temporal Cortex
infO	Inferior Occipital Cortex
infP	Inferior Parietal Cortex
infT	Inferior Temporal Cortex
MCC	Middle Cingulate Cortex
medF	Medial Frontal Cortex
medF_Orb	Medial orbital Frontal Cortex
midF	Middle Frontal Cortex
midO	Middle Occipital Cortex
midT	Middle Temporal Cortex
orbF	Orbital Frontal Cortex
paraHippo	paraHippocampus
PCC	Posterior Cingulate Cortex
postC	postCentral cortex
preC	preCentral cortex
RolOper	Rolandic Operculum
SMA	Supplementary Motor Area
supF	Superior Frontal Cortex
supO	Superior Occipital Cortex
supP	Superior Parietal Cortex
SupraMarg	supraMarginal gyrus
supT	Superior Temporal Cortex
